# A narrative review of the impact of interventions in acute kidney injury

**DOI:** 10.1007/s40620-017-0454-2

**Published:** 2017-11-29

**Authors:** Lynne Sykes, Rob Nipah, Philip Kalra, Darren Green

**Affiliations:** 1 Emergency Assessment Unit, Salford Royal NHS Foundation Trust, MAHSC, Stott Lane, Salford, M6 8HD UK; 2 Division of Cardiovascular Sciences, University of Manchester, MAHSC, Manchester, UK; 3 Department of Renal Medicine, Salford Royal NHS Foundation Trust, MAHSC, Salford, UK

**Keywords:** Acute kidney injury, Review of impact, AKI bundle, AKI nurses, E-alerts, Sick day rules

## Abstract

Acute kidney injury (AKI) is independently associated with significant morbidity and mortality, and is thus an important challenge facing physicians in modern healthcare. This narrative review assesses the impact of strategies employed to tackle AKI following the 2009 NCEPOD report on acute kidney injury (Sterwart et al. Acute kidney injury: adding insult to injury, pp 1–22, 2009). There is scarce and heterogeneous research into hard end points such as mortality and AKI progression for AKI interventions. This review found that e-alerts have varying effects on mortality and AKI progression, but decrease the incidence of contrast-induced AKI. The use of AKI bundles delivers statistically significant improvements in mortality and AKI progression. Similarly, AKI nurses generate statistically significant improvements on hospital acquired AKI and mortality. As yet there is no evidence base for the effects of education, sick day rules and smart phone apps. Overall, a combination of e-alerts and AKI bundles supported by education yielded the most effective and statistically significant results. Current practice revolves around reactive rather than preventative behaviour. This narrative review discusses reactive interventions and their impact on the progression and severity of AKI, and on mortality from it. Preventative behaviour, such as risk stratification and early intervention in the deteriorating patient, may be influential in decreasing AKI incidence.

## Introduction

### Overview

Acute kidney injury (AKI) is an important challenge facing physicians in modern healthcare. AKI is a common and serious syndrome present both in the community and in hospital populations. It is characterised by an acute deterioration in renal function and classified into Stages 1, 2 and 3 as shown in Table 1. A US single centre study of more than 15,000 emergency admissions to hospital found that AKI accounted for more than 1 in 5 of the presentations [2]. In a United Kingdom single centre study, 65% of AKIs identified had commenced in the community [3]. Specific sub-groups of patients are at particularly high risk of AKI, such as the elderly and those with pre-existing CKD. AKI is independently associated with significant morbidity and mortality, with a mortality of 23.9% in adults (95% CI, 22.1–25.7) shown in a 2013 meta-analysis [4]. AKI is linked with significant healthcare costs, [5] with ‘the cost of ignoring AKI’ priced at £1.2 billion in the UK [6].

**Table 1:** KDIGO acute kidney injury classification

AKI	Serum creatinine criteria	Urine output criteria
Stage 1	Increase of more than 0.3 mg/dl (≥ 26.4 µmol/l) or increase of 1.5 to twofold from baseline	< 0.5 ml/kg per hour for 6–12 h
Stage 2	Increase two to threefold from baseline	< 0.5 ml/kg per hour for > 12 h
Stage 3	Increase threefold or serum creatinine of more than or equal to 4.0 mg/dl (> 354 µmol/l) or initiation of renal replacement therapy	Less than 0.3 ml/kg per hour or anuria for > 12 h

This narrative review focuses on patient outcome of interventions employed to tackle AKI through changes in both investigation and management. A narrative review has been conducted because the lack of high quality studies for each intervention, combined with the heterogeneity of both study design and population, makes it difficult to produce a reliable systematic review or meta-analysis. This review will focus on studies since the seminal NCEPOD (National Confidential Enquiry into Patient Outcome and Death) report of 2009 [7].

### NCEPOD report 2009: ‘adding insult to injury’

The NCEPOD report ‘adding insult to injury: a review of the care of patients who died in hospital with a primary diagnosis of acute kidney injury (acute renal failure)’ in 2009 was chosen as a cut-off for review, as it was a milestone in the recognition of AKI, elevated its profile, and was a factor in the increase in studies of AKI interventions in the United Kingdom in recent years. This report stressed several key concerns in recognition and investigation of AKI, and highlighted poor adherence to basic clinical investigation protocols in a consistent and timely fashion. It emphasised that less than 50% of care was deemed good, while 43% of patients with hospital-acquired AKI had an unacceptable delay in recognition. Overall, the panel felt that there was poor recognition of sepsis, acute illness, and hypovolaemia. They concluded that 17% of hospital-acquired AKI could have been avoided. Underlying this, they described a failure to complete basic investigations and continue baseline physiological monitoring [7].

### Interventions post-NCEPOD

Several interventions have been developed with the aim of achieving significant improvements in the care of patients in hospital to both prevent and detect AKI, and to focus on swift management after identification.

**Fig. 1:**
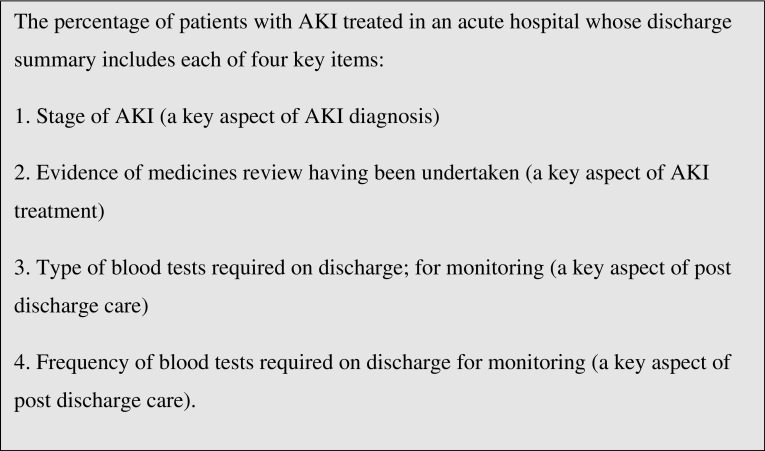
CQUIN indicators 2015/2016 for acute kidney injury

In response to the NCEPOD report, the commissioning for quality and innovation (CQUIN) instigated financial rewards for improving AKI care. In 2015/16 NHS England supported CQUIN’s financial rewards strategy by allowing NHS commissioners to offer such rewards to healthcare service providers under the NHS standard contract, providing the indicators detailed in Fig. 1 were complied with. Previously, when the advancing quality alliance (AQuA) pursued a similar strategy to focus attention on pneumonia, they found that a 6% reduction in mortality was achieved, as well as a saving of £10 for every £1 spent [8].

### Aims of this review

The pursuit of these CQUIN targets has led to several developments in the race to improve recognition of AKI and the ways in which healthcare professionals are alerted to investigate and initiate management. The aim of this review was to look at whether the specific interventions of electronic alerts (e-alerts), AKI nurses, AKI bundles, AKI apps for electronic devices, education, and sick day rules have improved outcomes for patients with AKI.

## Review method

A preliminary scoping exercise was undertaken. It indicated that the studies available were too heterogeneous to permit a systematic review or meta-analysis of the interventions developed to tackle AKI. Therefore a narrative review of the literature from January 2009 to November 2016 was undertaken using computer and internet databases. The review focussed on the period following 2009, since it was in that year that AKI started to attract significant media and medical attention and was pushed to the forefront of the national agenda following publication of the NCEPOD report.

The databases searched were NHS evidence, CINAHL, EMBASE, Medline and PubMed, Google Scholar and the Cochrane Library. There was an additional review of relevant references in the selected final papers. Studies were selected that considered adult patients only, had a defined intervention (AKI bundle, AKI nurse, e-alert, sick day rules, education package, AKI app), and a measured outcome (mortality, renal morbidity, change in creatinine, dialysis, AKI progression, AKI incidence).

The specific search keywords used are shown in Table 2, below. Each vertical column was combined using the Boolean operator OR. Each vertical column group was then combined using AND with the AKI column group.

**Table 2:** Key words used as Boolean operators or in search for articles

AKI	E-alert	Specialist nurse	AKI bundle	AKI app	Sick day rules	Education
Acute kidney injury	Electronic alert	Nurse	Bundle	Application	Sick day	Education package
Acute renal failure	E alert	Outreach		App	Sick day cards	Teaching
Acute renal impairment	Electronic flag			Smartphone	Sick day guidance	
ARF	Alert			Smart phone		

Papers were initially screened by title. At this stage duplicates and unrelated papers were excluded. After this initial refinement, the papers were then reviewed by abstract to determine relevance. All study designs were eligible. Finally, the full paper was reviewed and judged against the following inclusion criteria:

- Exclusively considered adults over the age of 18
- Rooted in secondary care, hospital only
- Written in English, from any country
- Published 2009–2016, in full and peer reviewed
- At least one AKI intervention (e-alert, specialist nurse, education package, AKI bundle, AKI app)
- At least one acute kidney injury outcome measured from the following—mortality, renal morbidity or change in creatinine or dialysis, AKI progression, AKI incidence.

**Fig. 2:**
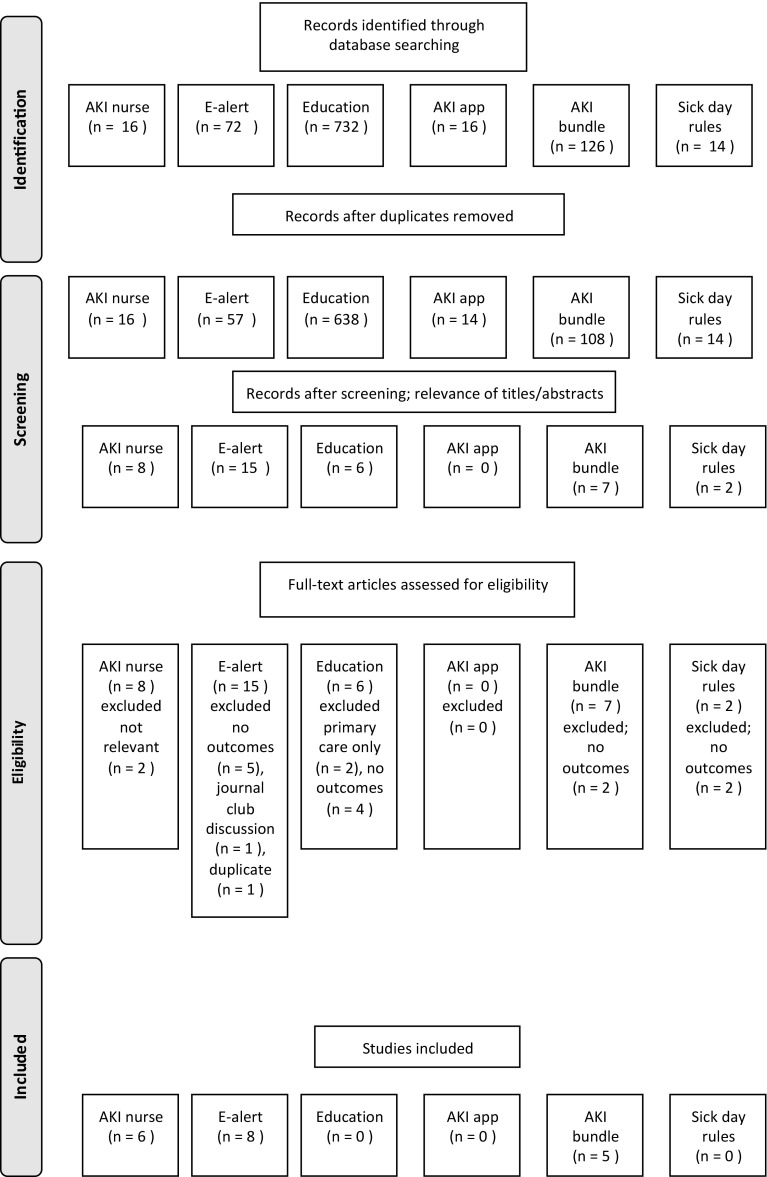
Number of articles meeting the criteria for inclusion by category

The total number of articles related to AKI and each intervention at each stage of the review process are found in Fig. 2.

The criteria were decided upon prior to the review being undertaken to create a robust framework while reviewing articles, and to ensure this review aligned with future research work that is planned by the department.

The main reasons for exclusion were concerned with no measured outcome related to AKI progression, mortality or incidence. The majority of seemingly relevant studies were excluded during review of the paper as they focussed on compliance with the intervention, rather than the effect the intervention had on AKI. Another significant section of papers covered epidemiological aspects of AKI that were generated from the advent of the e-alert.

## Results

### E-alerts

The introduction of the mandatory e-alert system has standardised criteria for AKI staging with a national algorithm for detection. This was established by NHS England in March 2015 and rolled out over the following year across primary care [5].

There have been numerous heterogeneous studies on the topic of e-alerts and their impact, generating a slowly growing body of evidence. As far back as 1994 Rind et al. [9] laid foundations for the current national algorithm with software that tracked creatinine, for over 1500 episodes of AKI, and sent an alert to the email of the responsible physician. This improved average the average time from change in creatinine to change in nephrotoxic medication by 21.6 h (p = 0.0001) with a risk of serious renal impairment of 0.45 (95% CI 0.22–0.94) when compared to the control period.

Table 3 summarises the e-alert studies included in this review. A single centre study in Belgium by Colpaert et al. found an increase in the number of early therapeutic interventions, (28.7% in e-alert group vs. 7.9 and 10.4% in the pre- and post e-alert control groups, respectively, p < 0.001). In the e-alert group, more patients received fluid therapy (23.0 vs. 4.9 and 9.2%, p < 0.01), diuretics (4.2 vs. 2.6 and 0.8%, p < 0.001), or vasopressors (3.9 vs. 1.1 and 0.8%, p < 0.001). However there was no change in length of stay in ICU, mortality, or severity of AKI [10]. This highlights balancing factors: the negative impact that interventions can have such as an increased workload or increased interventions with no related clinical improvement.

**Table 3:** Studies showing the effect of e-alerts on outcomes in AKI

Study	Number of patients	Setting	Outcome	Comment
Mortality				
Colpaert [10]	951 patients (e-alert control group 227; Alert group 616; Post alert control group 236)	ICU	No effect on mortality; mortality p = 0.37 Increase in early 28.7% in e-alert group vs. 7.9% and 10.4% in the pre- and post e-alert control groups, respectively, p ≤ 0.001	AKI with DECT phone alert, effect of AKI sniffer disappeared post intervention
Thomas [12]	157 pre intervention 251 post intervention	Hospital	No effect on mortality at 4 years	Intervention; e-alert. Initial 6% improvement in survival of post intervention group
Wilson [11]	1192 usual care 1201 intervention	Hospital	No effect on mortality [Odds ratio 1.16 (0.81–1.68); p = 0.40]	Intervention; pager alert for AKI with link to website
Ebah [19]	Number not declared, Quality improvement project; interventional before and after study	Hospital	Trend towards lower mortality 34 per month, vs 38 per month prior to intervention	Care bundle, AKI nurse, education
Selby [17]	8411 post alert, CB, education	Hospital	Decreased mortality p = 0.006	Unadjusted survival at 30 days improved from 76.3 to 80.5% over 6 months
Kolhe [18]	1209 pre alert 1221 post alert with CB	Hospital	Decreased in-hospital mortality p = 0.046	Mortality benefit persisted at 30, 60 and median follow up of 134 days for those with CB completed within 24 h of AKI
Chandrasekar [20]	Quality improvement project interventional study	Hospital	23.2% reduction in in-hospital mortality 25.9% reduction in 30-day mortality sustained over 33 months	Combined with care bundle, AKI nurse, education
AKI progression, creatinine rise or dialysis incidence				
Colpaert [10]	951 patients (pre-alert control group 227; Alert group 616; Post alert control group 236)	ICU	No effect on AKI progression or dialysis incidence	AKI progression p = 0.09, dialysis incidence 0.68 More and earlier interventions for AKI with DECT phone alert, effect of AKI sniffer disappeared post intervention
Wilson [11]	1192 usual care 1201 intervention	Hospital	No improvement in AKI progression (p = 0.81) or the incidence of dialysis [Odds ratio 1.25 (95% CI 0.90–1.74); p = 0.18]	AKI progression p = 0.81, dialysis incidence [OR 1.25 (95% CI 0.90–1.74); p = 0.18]
Kolhe [18]	1209 pre alert 1221 post alert with CB	Hospital	Less AKI progression p = 0.01	
AKI incidence				
Chandrasekar [20]	Quality improvement project interventional study	Hospital	Decrease in AKI 3	Combined with care bundle, AKI nurse, education
Ebah [19]	Number not declared, Quality improvement project; interventional before and after study	Hospital	31% reduction in incidence of AKI (9–6.5% admission incidence) hospital acquired (28% reduction)	Care bundle, AKI nurse, education
Cho [16]	258 pre 205 post alert	Hospital	Reduced incidence of Contrast induced-AKI p = 0.02	More contrast prophylaxis, 55% post vs. 25% pre alert

*OR* odds ratio, *CB* care bundle, *CI-AKI* contrast induced AKI

Wilson et al. produced the largest study of e-alerts post NCEPOD. This single centre study from the USA screened 23,364 adult patients, randomly assigning 1192 patients to standard care and 1201 patients to the intervention arm. They found alerts to be ineffective at improving outcomes [11]. They described an alert system for the intervention arm that relied on paging an automated electronic alert to the medical provider and pharmacist for each for AKI within 1 h of the alert. This alert contained a hyperlink to a website of study information and the latest KDIGO AKI guidelines. There was a parallel control group who received standard care without an alert. Overall there was no change to the way AKIs were managed. The website was not visited more frequently and nephrology referrals were not significantly increased. There was no improvement seen in AKI progression (p = 0.81), the incidence of dialysis [odds ratio 1.25 (95% CI 0.90–1.74); p = 0.18] or mortality between the groups [odds ratio 1.16 (0.81–1.68); p = 0·40]. Most importantly, there was no improvement in survival. A similar, smaller, UK-based single centre study by Thomas [12] that relied on the automated national e-alert detection system found similar conclusions. There was a mean age of 70 and around 80% of those patients with alerts were admitted to hospital. The intervention involved the primary clinical care team receiving a phone call to advise them on AKI management. They detected an initial 6% improvement in survival with the intervention group. However, this was no longer statistically significant when followed up at 4 years (p = 0.38 log rank test). Thomas’ study differed from Wilson’s by including a follow-up phone call to the team after the automated e-alert. This additional phone call or interaction appears to have an influence on human behaviours and may be what drives e-alert success.

How an e-alert is communicated is important to its acceptance—the process by which a fact is considered valid and adopted into clinical practice. As such, weaknesses in the format of an alert and/or the method of its delivery may account for failures to translate alerts into action. Technological and human elements combine in a complex relationship in an e-alert. Several of those elements typically combine to affect an e-alert’s efficacy, including placement, impact, frequency or intrusiveness of alerts within the software. Another issue is a high incidence of deferring or overriding alerts.[13, 14] Human factors such as habituation, banner blindness and alert fatigue are all key influences. Phansalkar et al. describe large pressures on the NHS from organisation, reorganisation and time shortage [15].

E-alerts that were linked to an intervention have yielded positive outcomes in terms of AKI incidence, AKI progression and AKI mortality. A key example of a proactive, rather than reactive, intervention is by Cho et al. [17] in the context of adult in-patients in a single centre and contrast prophylaxis. Cho linked an interruptive e-alert for the physician to consider contrast prophylaxis at the time of CT request for all patients with an eGFR < 60 ml/min/1.73 m^2^. This intervention led to a significant reduction in contrast induced AKI (CI-AKI) (p = 0.02) with a significant increase in contrast prophylaxis prescription in the intervention group of 55 vs. 25% [16]. Selby published a cautiously optimistic observational assessment from service developments of 8411 patients from a single UK centre. He reported lower mortality with the combination of e-alert, care bundle and an education package. The unadjusted survival data at 30 days showed an improvement in survival from 76.3 to 80.5% over 6 months [17].

A propensity score-matched cohort single centre study of 2297 patients by Kolhe et al. that used a care bundle to support the interruptive e-alert also found a significant decrease in mortality (p = 0.046, OR 0.46–0.89) that persisted for up to 4 months in multivariable analysis. This had a hazards ratio of 0.77 for those patients with AKI bundles completed within 24 h [18]. None of the subsequent studies have long enough follow-up periods or sufficient long term data to prove sustained improvement in mortality. The notable difference between those studies that demonstrated positive outcomes appears to be the introduction of a care bundle or interruptive checklist alongside the e-alert. It is likely that this secondary element, alongside the inevitable rise in profile of the intervention with education and awareness, is creating a redundancy within the system that allows AKI to be more reliably identified and its treatment to be instigated earlier.

Ebah and Chandrasekar [19, 20] each conducted quality improvement projects using a variety of tools to improve the recognition, investigation and management of AKI. These two studies had similar interventions: AKI nurses, education, an AKI bundle and e-alerts. The significant differences were that Ebah’s Manchester team developed an e-alert that was highly sensitive—more so than the national algorithm—and the AKI nursing team worked to remove any false positives. This interventional, quasi-experimental, longitudinal, before-and-after study generated significant improvements in AKI incidence (313 average new cases reduced to 215 cases per month, 2.5% reduction as proportion of admissions), on hospital-acquired AKI (28% reduction) and on length of stay (22.1–17 days, 23% reduction). There was also a trend toward improvements in mortality (average 38 deaths per month to 34 deaths per month) [19].

Meanwhile, the Liverpool team under Chandrasekar used all of the above interventions and an additional risk prediction score that was in use prior to the improvement project. They saw an overall reduction in mortality rate (23.2% reduction for in-hospital mortality, 25.9% for 30 day mortality) sustained over 33 months, a reduction in AKI 3 and a reduction in length of stay (2.6 days) [19, 20].

Other improvements seen as a product of the e-alert system are medication and pharmacy orientated. Several studies (McCoy [14], Terrel [21], Claus [22] and Awdishu [23]) identified more appropriate dosing, increased use of contrast prophylaxis and improved rates and timeliness of medication. However, they did not evaluate the patient outcomes that are within the scope of this paper. Such interventions need further evaluation and may well have a clinically relevant impact for AKI.

It is important to recognise that while the e-alert is now mandatory for detection of AKI, the process of alerting the key staff to engage in clinical correlation remains flexible. The e-alert must therefore be appropriately supported with a tangible set of actions such as the AKI bundle, and buttressed with a dynamic and accessible programme of education, as described by Ebah and Chandrasekar in their differing but similarly effective quality improvement projects [19].

E-alerts may also have led to an increase in AKI detection and awareness. A study in Italy noted that up to 75% of chronic kidney disease (CKD) is missed or not coded for [24]. Whereas by comparison a study from two large district general hospitals in the UK, found that only 10.5% of AKI was missed. They found significantly more episodes of AKI missed in surgery, however those that were missed were less severe [25]. Hospital acquired AKI was also evaluated in a large Chinese study. They found that AKI under the surgical team was found in the younger, male patients with less comorbidity than medical patients. They also found mortality to be lower with 11% in surgery as opposed to 39% in medicine [26]. There is also an association found between mortality and the time to referral. 4296 patients with hospital acquired AKI in a Swiss Tertiary referral centre were analysed. Those patients with late referrals or non referrals to nephrology were associated with increased mortality and worse renal outcome [27]. Some of the improvements in outcomes seen in the care bundles may have been influenced by the recommendation to refer.

### AKI care bundles

**Fig. 3:**
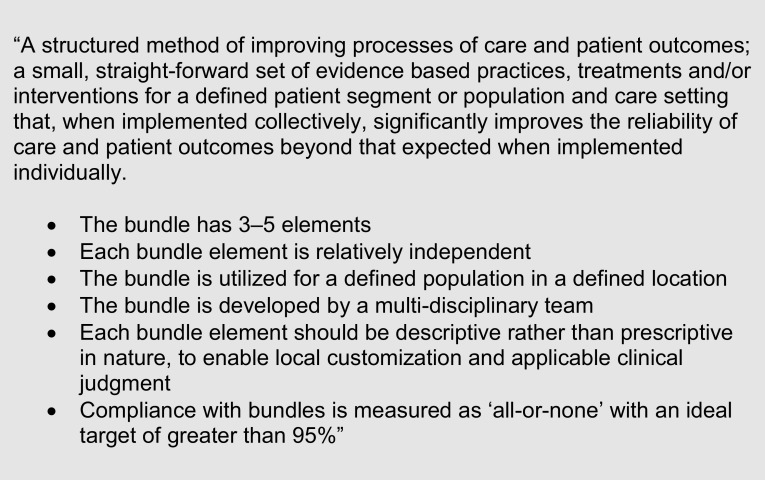
The International Healthcare Institute (IHI) definition of a ‘care bundle’ [45]

A care bundle is a collection of interventions grouped together to investigate and manage a specified condition. The International Healthcare Institute (IHI) definition of a ‘care bundle’ is shown in Fig. 3.

The rationale for the use of care bundles is clear from the track record laid down by the ‘Sepsis Six’ campaign and the sepsis bundle. Introduction of the sepsis bundle has halved mortality (21.2–8.7%) in a multicentre observation US cohort of 4329 patients; this was correlated with increased bundle compliance (4.9–73.4%) [28]. However, for AKI, rates of implementation and bundle completion remain low. Nguyen, in a prospective cohort study of 556 patients from eight tertiary medical centres in Asia [29], noted that bundle compliance improved from 13% (baseline) to 54% following education and four cycles of quality improvement work (p ≤ 0.01). Bundle completion equated to a relative risk reduction of death of 0.251 (95% confidence interval; 0.007–0.442). Steinmo [30] interviewed 34 medical professionals to explore barriers and influences on bundle compliance, allowing behavioural science to feed back into PDSA cycles and solve real-world care bundle application issues. There is a need to understand this phenomenon of improved outcome with relatively poor bundle completion compliance.

There is a growing body of evidence for the impact of the AKI care bundle that is summarised in Table 4. The bundles that had an impact and are included in this review contained five core elements. All of the bundles included consideration of the cause of AKI and investigation it. The next most common elements were a fluid assessment, urine dip and a referral to the renal team. Table 4 shows the outcome of the reviewed literature.

**Table 4:** Studies showing the effect of care bundles on AKI outcomes (adapted from Selby [43])

Study	Size/type	Setting	Bundle	Outcome
Mortality				
Kolhe et al. [18]	1209 pre CB 1291 post CB	Hospital	6 elements (fluid assessment, urinalysis, diagnose cause of AKI, order investigations, initiate treatment, refer)	Lower mortality p = 0.045, lower progression of AKI 1–2/3 p = 0.02
Ebah [19]	Quality improvement project; interventional before and after study	Hospital pilot 1 ward, scale up 4 wards then hospital wide	10 point Priority care checklist (baseline, cause, fluid assessment, cause and investigations, catheter, USS, renal referral, fluid balance, urine dip, drug review)	Trend towards lower mortality 34 per month, vs 38 per month prior to intervention
Chandrasekar [20]	Quality improvement project interventional study	Hospital	ABCDE-IT (Acute complications, Blood pressure, Catheterise, Drugs, Exclude obstruction, Investigations, Treat cause)	23.2% reduction in in-hospital mortality 25.9% reduction in 30-day mortality sustained over 33 months
Kolhe et al. [43]	3518 (939 with CB, 1823 without)	Hospital	6 elements (fluid assessment, urinalysis, diagnose cause of AKI, order investigations, initiate treatment, refer)	Lower mortality (20.4 vs. 24.4%, p = 0.017)
AKI progression, creatinine rise or dialysis incidence				
Tsui et al. [31]	55 patients pre and 53 post CB	Hospital	11 elements (baseline creatinine, fluid status, urinalysis, med review × 2, u PCR, urine output, renal USS, referral × 3)	Reduction in RRT in ICU 1.8–0% Reduction in HDU p ≤ 0.001, better documentation p ≤ 0.001
Kolhe et al. [43]	3518 (939 with CB, 1823 without)	Hospital	6 elements (fluid assessment, urinalysis, diagnose cause of AKI, order investigations, initiate treatment, refer)	Less AKI progression (4.2 vs. 6.7%, p = 0.02)
Chandrasekar [20]	Quality improvement project interventional study	Hospital	ABCDE-IT (Acute complications, Blood pressure, Catheterise, Drugs, Exclude obstruction, Investigations, Treat cause)	Weak inverse correlation of AKI incidence (R^2^ 0.351), decrease in AKI 3 and decrease length of stay (2.6 days)

*OR* odds ratio, *CB* care bundle, *u PCR* urine protein: creatinine ratio, *RRT* renal replacement therapy

Tsui et al. published their single centre UK audit results of 108 patients that focused on educating junior doctors to complete the bundle. This served to improve documentation (p  ≤ 0.001) with a reduction in high dependency unit admissions (p  ≤ 0.001) and renal replacement therapy in the Intensive Care Unit (1.8–0%) [31]. This study did not include hospital-acquired AKI, and acknowledged that junior doctors’ documentation was insufficient to ensure adequate completion of the bundle.

The prospective observational study of over 2000 adults in the UK carried out by Kolhe et al. [18] found that timeliness was a significant factor in outcomes. The authors assert that completion of a care bundle within 24 h of admission was associated with a significantly lower hazard ratio of death 0.771 (95% CI 0.620, 0.958) after a median follow-up of 134 days in comparison to those who did not have a care bundle completed within 24 h (p = 0.019).

As discussed in the previous e-alert “Results” section, Ebah and Chandrasekar [19, 20] both used care bundles as part of their quality improvement project interventions. Bundle compliance was considered as part of the discussion in each study. Ebah in particular considers the individual elements and their compliance in an “unbundled” analysis. If the ten elements of Ebah’s bundle were “unbundled” there would be 90% compliance, as compliance with urine dipstick was poor [19]. Chandresekar, however, did not analyse compliance with the care bundles as part of the quality improvement project [20].

Does compliance necessarily equate to improvement? Bhagwani’s quality improvement project of an AKI sticker, educational intervention and AKI bundle, audited 92 patients and found that 62% had a fluid chart pre-AKI bundle. Compliance actually decreased with bundle introduction. This result was thought by the researchers to reflect the isolated education given only to junior doctors and not the wider hospital staff, such as the nurses, who complete the fluid balance charts. Availability, awareness and accessibility of the physical bundle sticker also limited its use and the documented results [32].

Joslin et al. audited 192 episodes of AKI care at a Central London hospital and found significant improvements in recognition, fluid assessment and nephrotoxic cessation (all p ≤ 0.001) following introduction of their 8-element AKI bundle, but this was not correlated with improved patient outcomes [33]. Educational campaigns raise staff awareness, but significant complex external and human factors influence completion of bundles. As seen with the sepsis campaign, there is a constant need to assess and overcome barriers to implementation of the bundle to allow true evaluation of its impact [34].

### Educational packages

There is little research concentrating on the effect of education on outcomes in AKI, and none of it met the inclusion criteria for this review. Ebah and Chandrasekar each credit education as a contributor to the results seen in their respective quality improvement projects, with Ebah referring to a well-received and effective 4-slide headline tool [19, 20].

Gang Xu et al. have completed a two centre UK-based study looking at an educational package to improve outcomes in AKI. There were 319 questionnaires completed by physicians pre-intervention and 138 post-intervention. Their work improved awareness of AKI guidelines from 26 to 64% (p  ≤ 0.001), self-reported diagnosis of AKI (50 vs. 68%, p  ≤ 0.001) and investigating AKI (48 vs. 64%, p = 0.002) [35].

It is difficult to discern individual educational packages’ effects or impacts in isolation from other interventions, as it is implicit that a change such as an e-alert would require supporting information and education. Selby [17] maintains that the effect lies in a triad of strategies:

1. Detailed, bespoke education
2. Electronic detection and e-alerts
3. Care bundle.

### AKI nurses and AKI outreach teams

Different approaches to responsibility for AKI are adopted in different centres, with some considering AKI the responsibility of nephrologists, whereas others consider AKI to be everyone’s problem [3]. AKI specialist nurses are a growing factor in the interventions developed to tackle AKI. The nurses can provide targeted education to those wards with high prevalence in an opportunistic manner and create a redundancy in the system so that patients with AKI are not missed.

Thomas [12] described a phone call-based outreach service in a single UK centre (as discussed in the e-alert “Results” section) which, overall, generated more recommendations but garnered no statistical improvements. There is a delicate balance between improved AKI outcomes and increased work for radiology, nephrology and pathology colleagues. Gulliford [36] whose work covers three district general hospital settings within the UK, saw an increase in renal USS, renal review and senior review, but also saw better medication prescribing, less AKI 3, decreased LOS and decreased mortality.

Royal Liverpool University Hospitals state that since the introduction of an AKI team there has been a reduction in AKI progression and an 18% reduction in median hospital mortality. This has been partially achieved by combining an outreach team review for medically unwell patients with a bleep system for those scoring on the early warning system and prompt intervention and review [37].

The MAKIT better study [38] and Ebah [19] at the Central Manchester Foundation Trust both describe how the introduction of two AKI nurses led to improvements in several of the key areas. Ebah’s study is described in the “Results” section and the MAKIT better study saw similar results with regards to AKI incidence (18% reduction), hospital acquired AKI (1% reduction), mortality (10% reduction) and length of stay (10% reduction), although it is not stated whether these were statistically significant.

These appear to be showing a trend towards improvement. It may be that a combination of dedicated nurse time, “an extra pair of hands” assistance by outreach to give timely intervention, education and human interaction is more persuasive than an inanimate e-alert.

The key features of successful nurses or outreach teams where specified, were that they consisted of a specialist nurse with access to a consultant, either acute medicine or renal, and they operated 5 days a week between the hours of 9 a.m. and 5 p.m. The interventions were either ward visits or phone calls, with more favourable results linked with ward visits. A summary of these findings is found in Table 5.

**Table 5:** Studies showing the effect of AKI nurses and AKI outreach teams on AKI outcomes

Author	Intervention	Outcome
Thomas [12]	Outreach service	More recommendations made, initial 6% improvement in mortality, no statistical improvements long term
Hill [44]	AKI/outreach team-review AKI 2/3 and EWS scores > 5	Less AKI progression, 18% reduction in median hospital mortality
CMFT MAKIT [38]	AKI nurses, e-alerts, education	Decrease hospital acquired AKI (−1%), decrease mortality (−10%)
Gulliford [36]	AKI nurse, education, AKI champions, telephone follow up	Less AKI 3, decreased mortality
Chandrasekar [20]	Outreach team/AKI nurse, care bundle, e-alerts, education	Weak inverse correlation of AKI incidence (R^2^ 0.351), decrease in AKI 3 and decrease length of stay (2.6 days)
Ebah [19]	AKI nurses, e-alerts, education, care bundle	Decrease in AKI incidence (9–6.5%), decreased length of stay (22.1–17 days), trend towards improvement in mortality

### Smartphone applications, AKI app

Smartphones are now almost ubiquitous throughout both the general public and medical professionals, allowing immediate access to information at the point of care. Despite several AKI related apps from London, Edinburgh, Salford (AKI care) and Leeds (RRAPID—sepsis based) there is no data yet on their effectiveness or impact. As this intervention remains isolated from the NHS IT services, it is likely that mostly it will serve as an educational and reference tool. With the advent of Google and DeepMind integration at the Royal Free in London we await analysis from projects that may lead to developments in the future.

### Sick day guidance

There is no published quantitative evidence or long-term data on sick day guidance and its impact on AKI outcomes. The hypothesis for sick day guidance is that reducing or omitting medications such as anti-hypertensives or diuretics during an intercurrent illness will lead to a reduction in AKI incidence or progression. However this hypothesis has struggled from its conception. The main issue is a lack of consensus between renal and other specialities as there is little evidence to support this intervention thus undermining confidence in the premise. Heterogeneous groups of patients sustain AKI. As such, no “one size fits all” message is suitable. This is the key point in the qualitative piece by Morris et al. exploring the implementation of sick day guidance in primary care in the North West of England [39].

Several studies clearly indicate that combination medication such as angiotensin-converting enzyme (ACE-inhibitors), diuretics and non-steroidal anti-inflammatories (NSAIDs) are a risk for AKI. Tomlinson [40] found an increased prevalence of AKI in those on ACE inhibitors and angiotensin receptor blockade (ARB) over a 4 year study period from an observational study of around 8000 general practices across the UK. Likewise, Lapi [41] performed a similar nested case-control study of over 487,000 patients, with 2215 episodes of AKI, and found that a triple combination of diuretics, ACE inhibitors and NSAIDs increased incidence of AKI (rate ratio 1.31, 95% CI 1.12–1.53). There are professional consensus opinions published by the collaborative Think Kidneys Board [42] yet overall there is a need for improved resourcing and evidence base [39].

## Discussion

The NCEPOD of 2009 has been a great motivator by creating improved public awareness of AKI, increasing its profile in the NHS, and by provoking the introduction of financial incentives. This narrative review supports the growing body of evidence that grouped interventions can create an impact on the progression and severity of, and mortality from, AKI. Overall success appears to be due to a combination approach of an e-alert and an AKI bundle, supported by overarching education and an AKI nurse to create a failsafe within the system.

- The e-alert must be timely and appropriately intrusive to trigger actions such as the completion of an AKI bundle.
- All healthcare workers, from healthcare assistants, nurses and doctors both undergraduate and postgraduate, should undergo AKI education with a focus on risk recognition, the unwell patient and task prioritisation.
- There must be a redundancy built into the system, be it AKI nurses or dedicated pharmacist review, to mitigate for human factors and ensure that alerts translate into action.

### Where does the AKI community look to next?

At present the system is entirely reactive. For example, e-alerts and care bundles only commence once the insult has happened. In order to reduce AKI incidence there is a need for a proactive element. Successful and reliable risk modelling for AKI, coupled with education and rapid recognition of the deteriorating patient, may well result in an impact on incidence.

NCEPOD’s report “adding insult to injury” [1] suggests that simple achievable change lies in ensuring that the basics of patient monitoring and investigations are completed, then escalated, in a timely and appropriate fashion. This would include identification of, and early intervention for, those at high risk of AKI [1]. On-going audit of AKI incidence would create the opportunity for targeted education. This will probably rely on further research and a public and health sector wide programme of education. Further research is needed into the departmental and hospital variation in management of AKI and the corresponding outcomes.

A separate key intervention concerns feedback mechanisms between secondary and primary care. Dissemination of information from in-hospital patient stays or visits, such as discharge summaries and clinic letters, must improve in both quality and consistency, as must corresponding coding practices in primary care. The most discernible predictive factor for AKI is having had one previously. A patient who has had an AKI already has composite risk factors for AKI recurrence. Flagging up each patient with an AKI on discharge for review of these risk factors in the community should trigger consideration of secondary prevention.

## References

[1] Sterwart J, Findlay G, Smith N, Kelly K, Mason M (2009) Acute kidney injury: adding insult to injury. National Confidential Enquiry into Patient Outcomes and Death (NCEPOD), pp 1–22. https://www.ncepod.org.uk/2009report1/Downloads/AKI_report.pdf. Accessed 23 July 2017

[2] Wang HE, Muntner P, Chertow GM, Warnock DG (2012). Acute kidney injury and mortality in hospitalized patients. Am J Nephrol.

[3] Selby NM, Crowley L, Fluck RJ, McIntyre CW, Monaghan J, Lawson N, Kolhe NV (2012). Use of electronic results reporting to diagnose and monitor AKI in hospitalized patients. Clin J Am Soc Nephrol.

[4] Susantitaphong P, Cruz DN, Cerda J, Abulfaraj M, Alqahtani F, Koulouridis I, Acute Kidney Injury Advisory Group of the American Society of Nephrology (2013). World incidence of AKI: a meta-analysis. Clin J Am Soc Nephrol.

[5] NHS England: Acute Kidney Injury (AKI) Programme (2014). https://www.england.nhs.uk/patientsafety/akiprogramme/aki-algorithm/. Accessed 23 July 2017

[6] Kerr M, Bedford M, Matthews B, O’Donoghue D (2014). The economic impact of acute kidney injury in England. Nephrol Dial Transplant.

[7] MacLeod A (2009). NCEPOD report on acute kidney injury-must do better. Lancet.

[8] Sutton M, Nikolova S, Boaden R, Lester H, McDonald R, Roland M (2012). Reduced mortality with hospital pay for performance in England. N Engl J Med.

[9] Rind DM, Safran C, Phillips RS, Wang Q, Calkins DR, Delbanco TL, Slack WV (1994). Effect of computer-based alerts on the treatment and outcomes of hospitalized patients. Arch Intern Med.

[10] Colpaert K, Hoste Ea, Steurbaut K, Benoit D, Van Hoecke S, De Turck F, Decruyenaere J (2012). Impact of real-time electronic alerting of acute kidney injury on therapeutic intervention and progression of RIFLE class*. Crit Care Med.

[11] Wilson FP, Shashaty M, Testani J, Aqeel I, Borovskiy Y, Ellenberg SS, Fuchs B (2015). Automated, electronic alerts for acute kidney injury: a single-blind, parallel-group, randomised controlled trial. Lancet.

[12] Thomas ME, Sitch A, Baharani J, Dowswell G (2015). Earlier intervention for acute kidney injury: evaluation of an outreach service and a long-term follow-up. Nephrol Dial Transplant.

[13] Chertow GM, Lee J, Kuperman GJ, Burdick E, Horsky J, Seger DL, Bates DW (2001). Guided medication dosing for inpatients with renal insufficiency. JAMA.

[14] McCoy AB, Waitman LR, Gadd CS, Danciu I, Smith JP, Lewis JB, Peterson JF (2010). A computerized provider order entry intervention for medication safety during acute kidney injury: a quality improvement report. Am J Kidney Dis.

[15] Phansalkar S, Edworthy J, Hellier E, Seger DL, Schedlbauer A, Avery AJ, Bates DW (2010). A review of human factors principles for the design and implementation of medication safety alerts in clinical information systems. J Am Med Inform Assoc.

[16] Cho Aj, Lee JE, Yoon JY, Jang HR, Huh W, Kim Y-G, Oh HY (2012). Effect of an electronic alert on risk of contrast-induced acute kidney injury in hospitalized patients undergoing computed tomography. Am J Kidney Dis.

[17] Selby NM (2013). Electronic alerts for acute kidney injury. Curr Opin Nephrol Hypertens.

[18] Kolhe NV, Staples D, Reilly T, Merrison D, McIntyre CW, Fluck RJ, Taal MW (2015). Impact of compliance with a care bundle on acute kidney injury outcomes: a prospective observational study. PLoS One.

[19] Ebah L, Hanumapura P, Waring D, Challiner R, Hayden K, Alexander J, Hutchison A (2017). A multifaceted quality improvement programme to improve acute kidney injury care and outcomes in a large teaching hospital. BMJ Open Quality 6(1):u219176.w7476 10.1136/bmjquality.u219176.w7476 PMC5457974 28607684

[20] Chandrasekar T, Sharma A, Tennent L, Wong C, Chamberlain P, Abraham KA (2017). A whole system approach to improving mortality associated with acute kidney injury. QJM Int J Med.

[21] Terrell KM, Perkins AJ, Hui SL, Callahan CM, Dexter PR, Miller DK (2010). Computerized decision support for medication dosing in renal insufficiency: a randomized, controlled trial. Ann Emerg Med.

[22] Claus BOM, Colpaert K, Steurbaut K, De Turck F, Vogelaers DP, Robays H, Decruyenaere J (2015). Role of an electronic antimicrobial alert system in intensive care in dosing errors and pharmacist workload. Int J Clin Pharm.

[23] Awdishu L, Coates CR, Lyddane A, Tran K, Daniels CE, Lee J, El-Kareh R (2016). The impact of real-time alerting on appropriate prescribing in kidney disease: a cluster randomized controlled trial. J Am Med Inform Assoc.

[24] Gentile G, Postorino M, Mooring RD, De Angelis L, Manfreda VM, Ruffini F, Quintaliani G (2009). Estimated GFR reporting is not sufficient to allow detection of chronic kidney disease in an Italian regional hospital. BMC Nephrol.

[25] Meran S, Wonnacott A, Amphlett B, Phillips A (2014). How good are we at managing acute kidney injury in hospital?. Clin Kidney J.

[26] Tang X, Chen D, Yu S, Yang L, Mei C, Consortium, on behalf of I. A. 0 by 25 C (2017). Acute kidney injury burden in different clinical units: Data from nationwide survey in China. PLOS One.

[27] Meier P, Bonfils RM, Vogt B, Burnand B, Burnier M (2011). Referral patterns and outcomes in noncritically ill patients with hospital-acquired acute kidney injury. Clin J Am Soc Nephrol.

[28] Miller RR, Dong L, Nelson NC, Brown SM, Kuttler KG, Probst DR, Intermountain Healthcare Intensive Medicine Clinical Program (2013). Multicenter implementation of a severe sepsis and septic shock treatment bundle. Am J Respir Crit Care Med.

[29] Nguyen HB, Kuan W, Batech M, Shrikhande P, Mahadevan M, Li C-H, ATLAS (Asia Network to Regulate Sepsis care) Investigators (2011). Outcome effectiveness of the severe sepsis resuscitation bundle with addition of lactate clearance as a bundle item: a multi-national evaluation. Crit Care.

[30] Steinmo SH, Michie S, Fuller C, Stanley S, Stapleton C, Stone SP, Walshe K (2015). Bridging the gap between pragmatic intervention design and theory: using behavioural science tools to modify an existing quality improvement programme to implement ‘Sepsis Six’. Implement Sci.

[31] Tsui A, Rajani C, Doshi R, De Wolff J, Tennant R, Duncan N, Penn H (2014). Improving recognition and management of acute kidney injury. Acute Med.

[32] Bhagwanani A, Carpenter R, Yusuf A (2014). Improving the management of acute kidney injury in a District General Hospital: introduction of the DONUT bundle. BMJ Qual Improv Rep.

[33] Joslin J, Wilson H, Zubli D, Gauge N, Kinirons M, Hopper A, Ostermann M (2015). Recognition and management of acute kidney injury in hospitalised patients can be partially improved with the use of a care bundle. Clin Med.

[34] Tarrant C, O’Donnell B, Martin G, Bion J, Hunter A, Rooney KD, Krumholz H (2016). A complex endeavour: an ethnographic study of the implementation of the Sepsis Six clinical care bundle. Implement Sci.

[35] Xu G, Baines R, Westacott R, Selby N, Carr S (2014). An educational approach to improve outcomes in acute kidney injury (AKI): report of a quality improvement project. BMJ Open.

[36] Gulliford S, Wilson S (2015) Improving Patient Safety and Reducing Harm through the Development of an Acute Kidney Injury Specialist Service at Wrightington, Wigan and Leigh NHS Foundation Trust. https://www.thinkkidneys.nhs.uk/aki/wp-content/uploads/sites/2/2015/08/Wrightington-Wigan-Leigh-Case-Study-Improving-Patient-Safety-and-Reducing-Harm1.pdf. Accessed 23 July 2017

[37] Hill L, Zacharia T, Hill C, Hine T, Ahmed S (2015). The usefullness of an electonic acute kidney injury (AKI) alert system for early diagnosis and intervention in hospitalised patients with AKI. Nephrol Dial Transplant.

[38] How Manchester’s acute kidney injury team (MAKIT) is driving improvement (2017). https://www.thinkkidneys.nhs.uk/aki/wp-content/uploads/sites/2/2016/11/Central-Manchester-Think-Kidneyscase-study.pdf. Accessed 23 July 2017

[39] Morris RL, Ashcroft D, Phipps D, Bower P, O’Donoghue D, Roderick P, Blakeman T (2016). Preventing acute kidney injury: a qualitative study exploring ‘sick day rules’ implementation in primary care. BMC Fam Pract.

[40] Tomlinson LA, Abel GA, Chaudhry AN, Tomson CR, Wilkinson IB, Roland MO, Payne RA (2013). ACE inhibitor and angiotensin receptor-II antagonist prescribing and hospital admissions with acute kidney injury: a longitudinal ecological study. PLoS One.

[41] Lapi F, Azoulay L, Yin H, Nessim SJ, Suissa S (2013). Concurrent use of diuretics, angiotensin converting enzyme inhibitors, and angiotensin receptor blockers with non-steroidal anti-inflammatory drugs and risk of acute kidney injury: nested case-control study. BMJ.

[42] Griffith K, Blakeman AC, Fluck T, Lewington R, Tomlinson SN, Tomson C (2015) Sick day rules in patients at risk of acute kidney injury: an interim position statement from the think kidneys board. https://www.thinkkidneys.nhs.uk/wp-content/uploads/2015/07/Think-Kidneys-Sick-Day-Rules-160715.pdf. Accessed 23 July 2017

[43] Selby NM, Kolhe NV (2016). Care bundles for acute kidney injury: do they work. Nephron.

[44] Hill L, Zacharia T, Hill C, Hine T, Ahmed S (2015). The usefullness of an electonic acute kidney injury (AKI) alert system for early diagnosis and intervention in hospitalised patients with AKI. Nephrol Dial Transplant.

[45] Resar R, Griffin FA, Haraden C, Nolan T (2012) Using care bundles to improve health care quality. IHI Innovation Series white paper

